# Graphene Oxide as a Nanocarrier for a Theranostics Delivery System of Protocatechuic Acid and Gadolinium/Gold Nanoparticles

**DOI:** 10.3390/molecules23020500

**Published:** 2018-02-24

**Authors:** Muhammad Sani Usman, Mohd Zobir Hussein, Aminu Umar Kura, Sharida Fakurazi, Mas Jaffri Masarudin, Fathinul Fikri Ahmad Saad

**Affiliations:** 1 Materials Synthesis and Characterization Laboratory, Institute of Advanced Technology (ITMA), Universiti Putra Malaysia, Serdang 43400, Selangor, Malaysia; 2 Pharmacology, Faculty of Basic Health Sciences, Bauchi State University, Bauchi 65, Nigeria; ameenkura@gmail.com; 3 Department of Human Anatomy, Faculty of Medicine and Health Sciences, Universiti Putra Malaysia, Serdang 43400, Selangor, Malaysia; sharida.fakurazi@gmail.com; 4 Department of Cell & Molecular Biology, Faculty of Biotechnology and Biomolecular Sciences, Universiti Putra Malaysia, Serdang 43400, Selangor, Malaysia; masjaffri@upm.edu.my; 5 Centre for Diagnostic and Nuclear Imaging, Faculty of Medicine and Health Sciences, Universiti Putra Malaysia, Serdang 43400, Selangor, Malaysia; ahmadsaadff@gmail.com

**Keywords:** GO nanosheets, gadolinium, protocatechuic acid, gold nanoparticles, diagnostics, anticancer, theranostics

## Abstract

We have synthesized a graphene oxide (GO)-based theranostic nanodelivery system (GOTS) for magnetic resonance imaging (MRI) using naturally occurring protocatechuic acid (PA) as an anticancer agent and gadolinium (III) nitrate hexahydrate (Gd) as the starting material for a contrast agent,. Gold nanoparticles (AuNPs) were subsequently used as second diagnostic agent. The GO nanosheets were first prepared from graphite via the improved Hummer’s protocol. The conjugation of the GO and the PA was done via hydrogen bonding and π–π stacking interactions, followed by surface adsorption of the AuNPs through electrostatic interactions. GAGPA is the name given to the nanocomposite obtained from Gd and PA conjugation. However, after coating with AuNPs, the name was modified to GAGPAu. The physicochemical properties of the GAGPA and GAGPAu nanohybrids were studied using various characterization techniques. The results from the analyses confirmed the formation of the GOTS. The powder X-ray diffraction (PXRD) results showed the diffractive patterns for pure GO nanolayers, which changed after subsequent conjugation of the Gd and PA. The AuNPs patterns were also recorded after surface adsorption. Cytotoxicity and magnetic resonance imaging (MRI) contrast tests were also carried out on the developed GOTS. The GAGPAu was significantly cytotoxic to the human liver hepatocellular carcinoma cell line (HepG2) but nontoxic to the standard fibroblast cell line (3T3). The GAGPAu also appeared to possess higher T1 contrast compared to the pure Gd and water reference. The GOTS has good prospects of serving as future theranostic platform for cancer chemotherapy and diagnosis.

## 1. Introduction

The discovery of graphene and graphene derivatives in the field of nanoscience and nanotechnology has attracted a great deal of research attention, this is because of their wide range of exceptional properties, including electrical, mechanical and thermal properties, to mention a few [1]. Although the extent of these properties vary from one graphene derivative to another, the basic properties of the carbon-based materials can be found in all graphene derivatives, this has made the application of the materials to become diverse. Graphene was discovered in 2004 by Geim and his team, which has earned them a Nobel Prize in Physics [2]. The derivatives of graphene are graphene oxide (GO) and reduced graphene oxide (rGO), which are classified based on the type of chemical alteration done on the structure of graphene material. Graphene and the derivatives are equally classified based on their number of layers (single layer, bi-layer and multilayer), which make them differ in their surface chemistry and dimensions [3]. 

For biomedical applications of the graphene derivatives, such as drug delivery and biosensing, GO is often the favorite amongst the others due to its carboxylic acid structure [4]. Just like graphene, GO is a two-dimensional layered nanomaterial with a high surface area to volume ratio. It differs from graphene mainly by its uncharged epoxide (O) and hydroxyl (OH) groups, which are located in the basal plane of the graphene-like nanosheets [4]. This enables them to have hydrogen bonding and other interactions at the layer surface. Other interactions, such as π–π and non-convalent bonding are also likely in the GO plane due to the presence of free unboned π electrons [5,6]. Unlike hydrophobic graphene, GO is hydrophilic and hence, more soluble and stable in colloids than its graphene counterpart [4]. In addition, GO has the ability to control drug release and it can be easily functionalized with other molecules. The aforementioned properties make GO a preferable nanocarrier in biomedicine, especially in drug and gene delivery systems.

Lately, GO has been used in bioimaging and theranostic applications. Theranostic systems involves the simultaneously delivery of therapeutic and diagnostic agents to the desired targets. Theranostic systems can be bimodal or multimodal, depending on the set-up of the delivery system. In bimodal mode, a therapeutic agent is loaded together with two diagnostic agents or a diagnostic agent with two imaging modalities on a nanocarrier, for drug delivery and imaging applications [7]. Theranostic delivery systems (TDS) are particularly of great interest in anticancer research. This is because cancer chemotherapy, which is the most utilized form of cancer treatment, involves diagnosis with sophisticated molecular imaging techniques, such as magnetic resonance imaging (MRI) and computed tomography (CT) [5]. Most of the imaging techniques require contrast agents due to the poor visibility in subject tissues; the contrast agents are mostly administered prior to the tests [8]. The TDS reduces the toxicities of these diagnostic agents as well as that of the toxic therapeutic agents by targeting the cancer cells [9]. In addition, TDS offers the possibility of monitoring the release of the cancer agents at the target site/cancer cells location [7]. MRI was used for the diagnostic aspect test in this work, while gadolinium and AuNPs were used as contrast agents for the MRI. 

Gadolinium is a trivalent rare earth metal that has been used in improving the contrast of MRI machines since 1984 [10], the contrast agents are used to increase the intensity of the signals generated in the MRI (T1 and T2). T1 is the spin–lattice relaxation signal and T2 is the spin–spin relaxation signal. Gold nanoparticles (AuNPs) are known for their superior surface properties, such as very high surface area to volume ratio and surface plasmon resonance (SPR) [11]. These properties among others make them useful in imaging applications. 

A handful of articles have reported GO-based nanoparticles for theranostic applications. The GO nanosheets were in most of the reports conjugated or functionalized with either polymers or ligands to improve their biocompatibilities [12,13]. In our work, no conjugating substance was employed for biocompatibility or adsorption of the guest molecules on the GO nanolayers. The GO nanosheets were prepared and directly loaded with firstly the MRI contrast agent (gadolinium (Gd)) by electrostatic interaction, which was trailed by doping of the anticancer agent (protocatechuic acid) through OH bonding and π–π stacking interactions. 

Protocatechuic acid (PA) is a natural compound found in medicinal plants, such as *Hibiscus sabdariffa* L. and *Ginkgo biloba* L. The compound is known for its several medicinal properties, including anti-hypertensive [14] anti-antibacterial [15], anti-inflammatory and analgesic [16], and anti-aging effects [17]. It has also been reported to have anticancer [14] properties. This influenced our choice of the drug as the anticancer drug in this work and as a replacement for the established toxic anticancer agents, such as doxorubicin (DOX) [12,13], which has been reported in various articles. In this work, GO nanosheets were used to conjugate the natural compound (PA) simultaneously with the Gd contrast agent (GAGPA). Subsequently, the GAGPA nanohyrid was used to adsorb AuNPs as the second MRI contrast agent (GAGPAu). GAGPAu is also referred to graphene oxide (GO)-based theranostic nanodelivery system (GOTS). Although most articles use AuNPs for CT contrast improvement, it was used as supporting MRI contrast in this work. 

## 2. Results and Discussion

**Figure 1:**
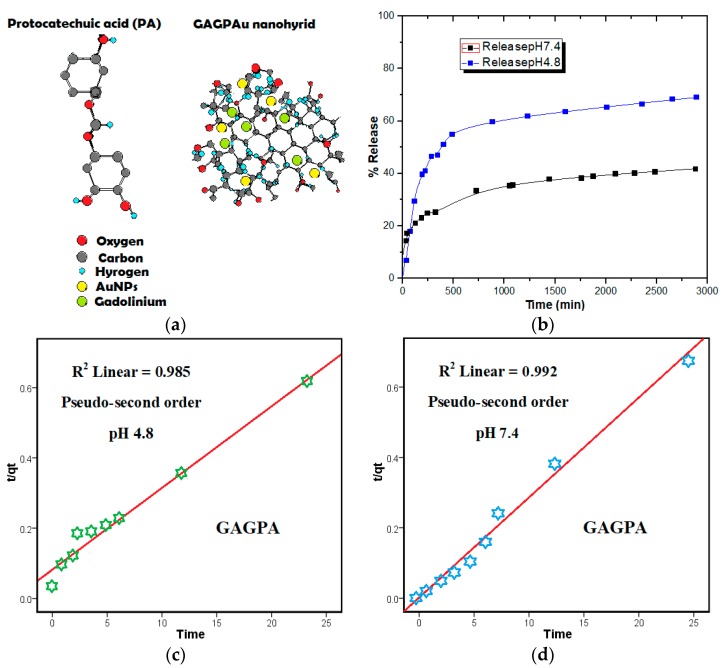
(**a**) Active agents-loaded of GAGPAu nanocomposite in a theranostic nanodelivery system. Diagnostic agents, gadolinium (green) and AuNPs (yellow) and the anticancer agent, PA are attached on a graphene sheet via hydrogen bond, π–π interaction and electrostatic interaction (GOTS); (**b**) Release profiles of protocatechuic acid from GO-Gd/PA nanocomposite (GAGPA) in pH 7.4 and 4.8 media; (**c**) Pseudo−seconder order kinetic plot of protocatechuic acid release data at pH 4.8 medium from GAGPA nanocomposite (Drug-GO/Gd); (**d**) Pseudo−seconder order kinetic plot of protocatechuic acid release data at pH 7.4 medium from GAGPA nanocomposite (Drug-GO/Gd).

This section highlights the results from the characterization of the pure phases and developed nanocomposites. Studies have shown that aromatic molecules mostly chemotherapeutics can be doped on the surface of GO nanosheets using their sp^2^-carbon sites as base for π–π stacking interactions or the COOH groups for hydrogen bonding with the guest molecules. The aforementioned interactions have been confirmed and meticulously discussed according to the methods of characterization. The GOTS is developed based on the concept of theranostic delivery system (TDS) with therapeutic and diagnostic agents both loaded on the GO layers. Figure 1a illustrates the theranostic arrangement of the GO nanosheets conjugated with protocatechuic acid and gadolinium, with AuNPs adsorbed on the surface in a typical TDS setting. 

### 2.1. Protocatechuic Acid Release Pattern from GAGPA Nanocomposite

Profiles of the drug release from GAGPA nanocomposite were obtained both in PBS media (pH 7.4 and 4.8). As shown in Figure 1b, the drug release started at around 5 min and stopped after about 3000 min, with over 65% of the drug was released in pH 4.8 and 40% in pH 7.4. This is understandably due to the gradual dissolution and detachment of the anticancer agent from the GO nanocarrier, which are bind by hydrogen-bonding and *π–π* stacking to the hydroxyl groups as well as the sp^2^ carbon atoms of the GO nanosheets [12,17]. Although based on the aromatic structure of the drug, the hydrogen bonding between the carboxylic and epoxide groups of GO and the hydroxyl groups of the protocatechuic [18] is favored in this case. The higher release process in the acidic medium than in the alkaline medium could also be due to the increase in hydrogen bonding propensity under acidic conditions, which would result in higher competition among the hydrogen bearing groups. This process weakens the hydrogen bonding between the carboxylic groups and the hydroxyl groups of the GO nanolayers and the protocatechuic acid respectively [5], hence the variation in the release profiles. The release in the alkaline medium could also be influenced by ion-exchange reaction [19]. The simulation release mechanism implies higher release in the actual cancer cells than in blood stream or non-cancer cells, since the host environment of cancer cells is acidic and vice versa for the blood and tissues. This has been established since 1960 by Ehrlich, where tumor-targeting is said to be based on the acidity of the pathological sites [20]. The release profiles are indicating the successful adsorption of the anticancer agent on GO nanosheets, which is in conformity with the anticancer evaluation of the nanocomposite as discussed in the cytotoxicity studies of this work. It is worthy of mentioning, that the percentage drug release profiles of our GAGPA nanocomposite is much higher than the reported drug release profiles of GO-conjugated/DOX nanocomposite [21]. 

#### Protocatechuic Acid Release Kinetics from GAGPA Nanocomposite

The kinetic release of protocatechuic acid from the GAGPA nanocomposite was studied for clear understanding of the drug release behavior. Three kinetic models were used to study the drug release:

(1) $$\mathrm{Pseudo-first}\;\mathrm{order}:\;\ln(q_{e}-q_{t})=\ln q_{t}-kt$$

(2) Pseudo-second order:  tqt=1kqe2+tq

(3) $$\mathrm{Parabolic}\;\mathrm{diffusion}:\;(1-M_{t}/M_{o})/t=kt^{-0.5}+b$$

In the above equations, $q_{e}$ and $q_{t}$ represent the amounts of PA released at equilibrium (*e*) and at time (*t*), while $M_{t}$ and $M_{o}$ are representing the amount of the PA in the nanocarrier at the time of release *t* and 0, respectively and *k* is the rate constant [22]. Although the drug release data was analyzed with the 3 models, only the best fit plot was presented in this paper (Figure 1c,d). However, the correlation coefficients (R^2^) deduced from all the three model plots are presented in Table 1. The pseudo-second order kinetic model was observed to be the best fit for the PA release data from the GAGPA nanohyrid, with correlation coefficients (R^2^) of 0.985 for pH 7.4 and 0.992 for pH 4.8. As reported in Table 1, the R^2^ of the first order is 0.863 (pH 7.4) and 0.563 (pH 4.8), while the parabolic diffusion is 0.936 (pH 7.4) and 0.932 (pH 4.8). Further, the rate constant (k) derived from the pseudo-second order model is 4.9 × 10^−3^ and 2.3 × 10^−3^ g/mg h for pH 7.4 and 4.8, respectively. Other information deduced from the model and its plot is the percentage saturation (%) and *t*_1/2_ (min), which have all been summarized in Table 1. 

### 2.2. Powder X-ray Diffraction Studies

**Figure 2:**
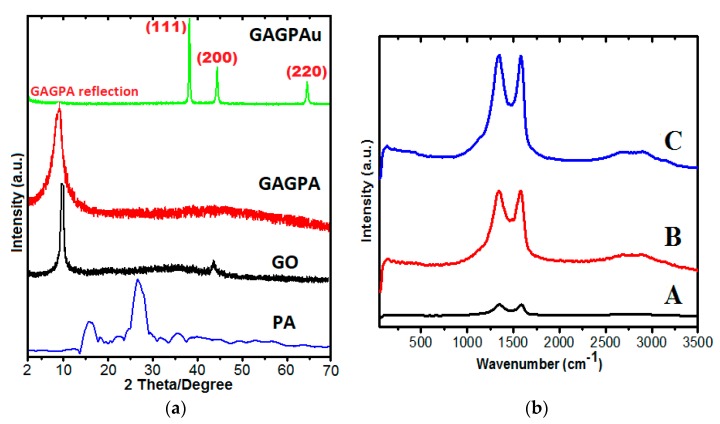
(**a**) PXRD diffractograms of the pure protocatechuic acid, GO nanosheets, protocatechuic acid loaded on GO/Gd nanolayers (GAGPA) and gold nanoparticles coated on GAGPA nanocomposite (GAGPAu); (**b**) Raman spectra of GO nanosheets (A), protocatechuic acid loaded on GO/Gd nanolayers (GAGPA) (B) and gold nanoparticles coated on GAGPA nanocomposite (GAGPAu) (C).

The step by step loading of the guest molecules into the GO nanocarrier was monitored by PXRD, in which the X-ray patterns were obtained at various stages. The patterns of the pure GO synthesized from the graphite material using improved Hummer’s method were obtained. Subsequently, the diffractograms of the pure drug was also taken and lastly the diffractograms of GO after Gd and protocatechuic acid loading (GAGPA) as well as after coating with AuNPs GO-Gd/PA-Au, as named GAGPAu in Figure 2a. The pure GO nanosheets XRD reflection can be seen at around 2θ position = 10° degrees (d = 8.5 Å) in the GO diffractogram [23,24], indicating successful preparation of the GO nanolayers. However, emphasis is on the GAGPA nanocomposite which was obtained after drug and Gd loading. The GAGPA diffractogram appears to have shifted a bit towards the lower 2θ angle on the plane, and broader in shape than the pure GO diffractogram. This is an indication of hydrogen-bonding and π–π stacking bonding between the carboxylic groups of GO [25] and hydroxyl groups of the drug as suggested by the drug release profiles [5,26,27]. It also could be due to electrostatic interaction between Gd and GO surface. The interactions appear to be at the surface only and not within the GO interlayer sheets. Generally, the PA and Gd loadings did not result in significant changes in phase in the GO nanosheet structure. Nevertheless, after coating with AuNPs (GAGPAu), the diffractogram assumes the diffractogram of the pure AuNPs. Only a weak reflection of the GAGPA can be noticed in the diffractogram. All other reflections are distinctive of the face centered cubic structure (FCC) of AuNPs (111, 200, 220, at 38°, 45° and 65° theta positions, respectively) [11]. This is due to the electrostatic interaction between the negatively charged GO surface and the positively charged AuNPs [12].

### 2.3. Raman Spectroscopy Studies

The degree of disorder that is induced by drug loading through hydrogen boding as well as surface coating with AuNPs was assessed through Raman spectroscopy using the Raman shift. The disorder peaks (D band) and graphitic peaks (G band) obtained from the pure GO and after successive loadings with the guest materials (GAGPA and GAGPAu) were studied to support other studies conducted on the GOTS and the nanocarrier. It can be seen in Figure 2b that the visible variations in the bands intensities of the samples at different levels of loading. The intensities of the bands appear to be increasing at every stage of modification, starting with GO nanosheets in Figure 2b (A). The intensity of the D and G bands are lower than the intensities of the D and G bands of the GAGPA nanocomposite (Figure 2b (B)). Consequently, the intensities of the D and G bands of GAGPA are lower than those of the GAGPAu nanocomposite (Figure 2b (C)). This is presumably due to the surface interactions that occurred between the GO and the PA molecules (mainly hydrogen bonding and π–π interactions), and subsequently between the GO nanosheets and the positively charged AuNPs (electrostatic interactions) [12]. 

In addition, the I_D_/I_G_ intensity ratios appear to be increasing slightly in the order of the surface activities. The relative intensity ratio of D to G bands (I_D_/I_G_) serve to indicate the degree of disorder/functionalization in a graphitic material [28] and are non-proportionate to the sp^2^-carbon clusters sizes [29]. The sp^2^-carbon atoms are believed to be the site of the π–π interactions [12]. Nevertheless, the estimated I_D_/I_G_ of the pure GO, GAGPA and GAGPAu nanocomposites are 0.84, 0.85 and 0.95, respectively. This slight increase after every stage of modification is also an indication of successive interactions at the various stages of the GO-based nanocomposite synthesis. Similar observations have been previously reported by other researchers who conjugated GO with other therapeutics in drug delivery applications [23]. 

### 2.4. Thermal Studies

**Figure 3:**
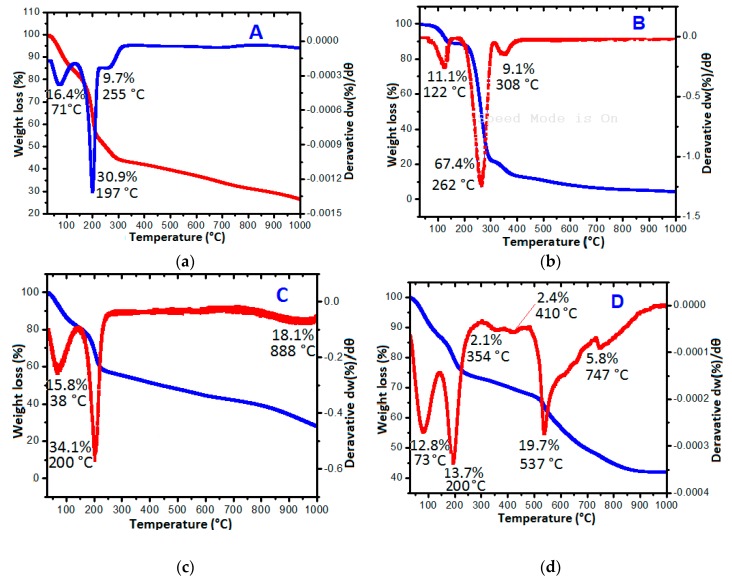
TGA (Blue) and DTG (Red) thermograms of (**a**) GO nanosheets; (**b**) pure protocatechuic acid; (**c**) protocatechuic acid loaded on GO/Gd nanolayers (GAGPA); (**d**) gold nanoparticles coated on GAGPA nanocomposite (GAGPAu).

The thermal changes of the GO nanolayers at different levels of modification were monitored by TGA/DTG analysis. The thermal activities were used to support other studies in the confirmation of the GOTS formation at various stages. The thermal decompositions of the pristine GO nanosheets were first studied to confirm the initial formation of the GO nanolayers from graphite source by the improved Hummer’s method. Consequently, the thermal studies of the pure PA, the GAGPA and GAGPAu nanocomposites were followed. Figure 3a–d and Table 2 present the thermograms of pure GO, pure PA, GAGPA and GAGPAu nanocomposites. Table 2 also highlights some of the important parameters associated with the thermal activities, which include, decomposition temperature range (T_range_), maximum peak temperature (T_max_) and change in mass [(decomposition mass) Delta m]. The GO thermogram (Figure 3a) did not show much activity, only three decompositions can be observed, starting with decomposition at 71 °C (16.4%) due to loss of water, second and the major decompositions at 197 (30.4%) is due to breakdown of the GO bonds, that is graphene to oxygen functional groups (reduction) and lastly the residue at 255 °C (9.7%) [25]. 

The protocatechuic acid thermogram in Figure 3b equally shows three decompositions, starting with the removal of absorbed water at 122 °C (11.1%) [26], and followed by PA decomposition at 262 °C (67.4%) [27]. The residue from PA decomposed at 308 °C (9.1%). The GAGPA thermogram in Figure 3c shows similar decomposition patterns as the drug and the GO. Although it appears more thermally stable than the GO nanosheets, which could be attributed to the conjugation that occurred as a result of hydrogen bonding between the GO functional groups and the drug. The major decomposition of GO appears at 200 °C (34.1%), which is slightly higher than in its pure form (197 °C). In addition, the PA residue decomposed at much higher temperature (888 °C, 18.1%) which is likewise higher than in the pristine PA (308 °C). Thus, GAGPA is considerably more stable than the pristine components. This has been observed by other researchers, where the developed composites appeared to be more stable thermally than the individual GO and the therapeutic agents, which confirms the hydrogen bonding between GO and the drug [5]. 

At the last stage of loading (after coating of AuNPs), the thermal events appear to be more than in the previous thermograms. Figure 3d is the GAGPAu thermograms, which indicates various decompositions that are linked to the individual loaded guests. The decompositions appear to be fragmented at different temperatures, nevertheless, the major decompositions are at 73 °C (12.8%) 200 °C (13.7%), 537 °C (19.7%) and 747 °C (5.8%) representing loss of physically-adsorbed water, GO decomposition, PA decomposition and AuNPs decomposition, respectively. 

The decompositions have confirmed the successive doping of the guest molecules onto the GO nanocarrier and thus, formation of GO-based TDS [9]. It is also comprehensible that GAGPAu nanocomposite is the most thermally stable amongst its counterparts.

### 2.5. Fourier Transformed Infrared Spectroscopy Analysis

**Figure 4:**
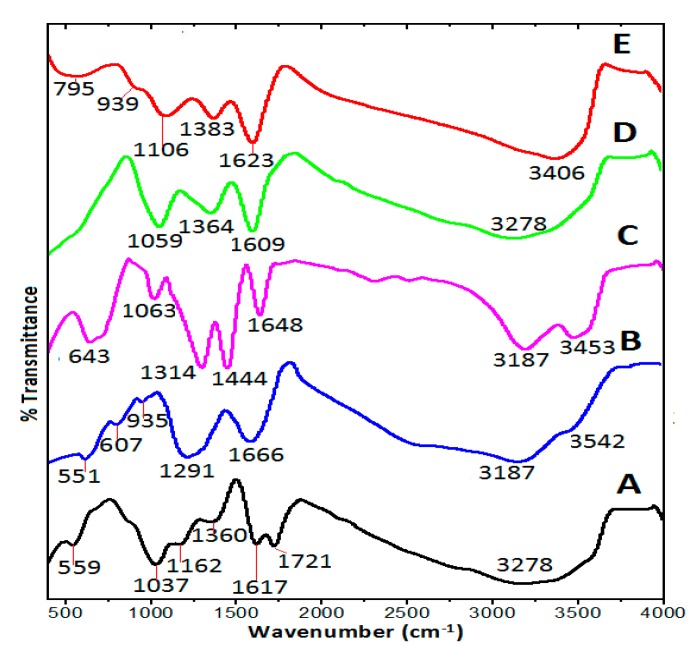
FTIR spectra of GO nanosheets (A); pure protocatechuic acid (B); pure Gd(NO_3_)_3_ (C) protocatechuic acid loaded on GO/Gd nanolayers (GAGPA) (D); and gold nanoparticles coated on GAGPA nanocomposite (GAGPAu) (E).

The chemical interactions between the GO nanosheets and the adsorbed species were further studied with a Fourier transform infrared spectroscopy (FTIR) analysis at various phases’ of preparation. The absorptions spectra of the pure GO, pure PA, pure Gd, drug and Gd loaded GO nanocomposite (GAGPA) and the GAGPA nanocomposite coated with AuNPs (GAGPAu) are presented in Figure 4A–E. The GO nanocarrier FTIR spectrum in Figure 4A depicts a broad and intense band of –OH stretching at 3278 cm^−1^, which is attributed to the hydroxyl groups that are present in the GO. The broadness of the peak could be due to bonding of the OH to carbon atoms present in the structure [30]. The stretching vibration of C=O bonds is observed at 1721 cm^−1^, the peak is ascribed to carbonyl and carboxylic acid groups of GO. The band for C=C bonds appeared at 1617 cm^−1^ and are linked to remnant of sp^2^ or unoxidized carbon structure of graphite [31]. The COH bonding is observed at 1360 cm^−1^ [30]. The C–O stretching vibrations can be seen at 1162 and 1037 cm^−1^, which are also attributed to oxidation of GO. In the PA spectrum (Figure 4B), a broad band at 3187 cm^−1^ is due to O–H stretching vibrations [32]. A band at 1666 cm^−1^ is assigned to C=C and the one at 1291 cm^−1^ is associated with the carboxyl group (C=O stretching) of protocatechuic acid [33]. The carboxylic group OH bending vibrations appeared between 935 and 551 cm^−1^. The Gd(NO_3_)_3_ spectrum is shown in Figure 4C; two bands at 3453 and 3187 cm^−1^ are linked to O–H stretching vibration [30], a band at 1650 cm^−1^ is associated with H_2_O bending vibration [34]. The two bands at 1444 and 1314 cm^−1^ are due to the NO_3_-stretching vibration of the nitrate group [35]. The GAGPA and GAGPAu nanocomposite spectra (Figure 4D,E, respectively) are similar to the GO spectrum. However, new bands and shift in bands can be observed when compared to the pristine GO, which are resulting from the surface interactions between GO and the loaded compounds. For example, the stretching vibration of C=O band at 1721 cm^−1^ is missing in all the nanohybrids. This is due the hydrogen bonding between carbonyl group of the GO and the PA hydrogen bearing groups.

Further, the C=C absorption band has shifted to 1609 and 1623 cm^−1^ and has become sharper and stronger in the GAGPA and GAGPAu, respectively. This is presumably due to π–π interactions, which usually occur at the sp^2^ carbon [36]. Likewise, the COH bonding absorptions can be seen to have shifted to 1364 and 1383 cm^−1^ and even stronger in the GAGPA and GAGPAu, respectively. The C–O stretching vibrations have also shifted to 1059 and 1106 cm^−1^ in the GAGPA and GAGPAu, respectively. These are all indications of surface interactions as observed in the Raman shifts of the pure GO and the nanocomposites. 

### 2.6. Transmission Electron Microscopy Studies

**Figure 5:**
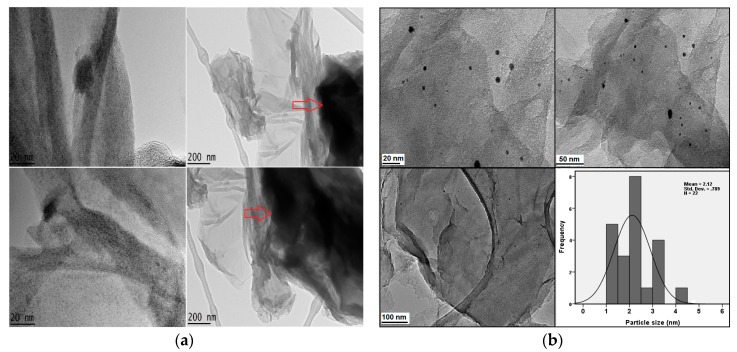
(**a**) TEM micrographs of protocatechuic acid loaded on GO/Gd nanocarrier (GAGPA) at high and low magnifications; (**b**) TEM micrographs of protocatechuic acid loaded on GO/Gd nanocarrier after surface coating with gold nanoparticles (GAGPAu).

The micrographs of the nanocomposites were taken after drug loading and AuNPs coating, GAGPA and GAGPAu, respectively. The purpose of this study is to understand the shapes, sizes and to certain extent the morphology of the developed nanohybrids. Although transmission electron microscopy (TEM) is used in the determination of shapes and sizes, the general morphology can be viewed at low magnifications micrographs. Figure 5a,b are micrographs of the GAGPA and GAGPAu nanocomposites at different magnifications. The GAGPA micrographs show typical multiple layered structure of GO with deposition of the drug on the surface [37]. The deposits of the drug can be seen in the micrographs with 200 nm magnification, as indicated by the red arrow. This confirms the earlier assertions from XRD, FTIR, Raman spectroscopy and TGA analyses that suggest the successful loading of PA on the GO nanosheets. 

On the other hand, the GAGPAu nanocomposite micrographs show predictable outcome, where the positively charged AuNPs can be seen to be adsorbed on the surface of the GO nanosheets. The adsorbed species are believed to be bonded onto the GO surface by electrostatic interactions. Nevertheless, the deposited AuNPs are spherical in shape and predominantly small in size. The mean average size of the AuNPs is around 2 nm, as deduced from the histogram and distribution curve. The results are in agreement with the XRD diffractograms, where pure AuNPs reflections were observed in the GAGPAu nanohybrid. Similar observation has been previously reported by Usman et al. [9], where layered double hydroxide (LDH)-based nanohybrid was surface coated with AuNPs. The micrographs also confirm the earlier assertion of successful formation of a GO-based TDS. 

### 2.7. Cytotoxicity Studies

Cytotoxicity studies were conducted to determine the effectiveness of the theranostic nanodelivery system of the anticancer agent. The tests determine the level of toxicity to healthy cells. In doing so, two cell lines were used in testing the cytotoxicity of the GO nanocarrier, the final TDS that is after AuNPs doping (GAGPAu) and the anticancer drug, pure PA. Standard fibroblast cell line (3T3) was used to test the safety of the TDS as to healthy cells, whilst human liver hepatocellular carcinoma cell line also known as HepG2, was used for cancer cytotoxicity test. 

**Figure 6:**
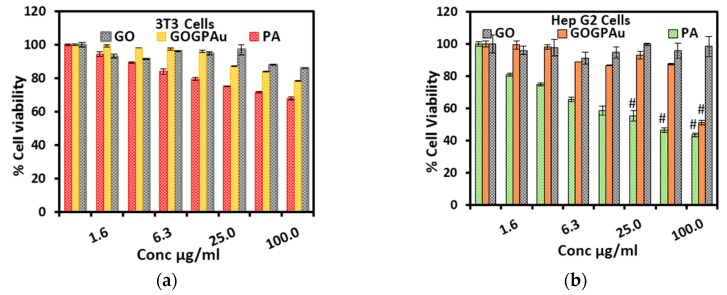
(**a**) Cytotoxicity results of pure GO nanosheets, pure protocatechuic acid and protocatechuic acid loaded on GO/Gd nanocarrier after surface coating with gold nanoparticles (GAGPAu) dosed in normal fibroblast cell lines (3T3); (**b**) cytotoxicity results of pure GO nanosheets, pure protocatechuic acid and protocatechuic acid loaded on GO/Gd nanocarrier after surface coating with gold nanoparticles (GAGPAu) dosed in cancer cell lines (HepG2).

Figure 6a,b show the data expressed in form of histogram obtained from the zones of inhibition of the study in 3T3 and HepG2 cell lines, respectively. The samples were dosed in various concentrations; 0.0, 1.6, 3.1, 6.3, 12.5, 25.0, 50.0 and 100.0 μg/mL for both cell lines. It is evident from the chart that the cells appear to have grown above average in all the concentrations, indicating the TDS, GO and the PA drug are non-toxic towards the 3T3 cells (Figure 6a) even at the highest dose which also suggests that they could be negligibly cytotoxic or nontoxic to healthy human cells. 

Contrary to the 3T3 cell lines, the HepG2 shows inhibited growth at 100 μg/mL concentration. The PA and the GAGPAu TDS have shown a high anticancer efficacy at 100 μg/mL, where the cancer cells are observed to show growth below average (Figure 6b). This implies that the GOTS could inhibit cancer growth. However, the GO nanocarrier did not indicate any efficacy even at the highest dose, since the cancer cells show almost 100% growth at 100 μg/mL GO dose. This also suggests the non-susceptibility of the cancer cells towards the GO nanosheets or the non-anticancer properties in the synthesized GO nanosheets. Similar results were reported by He et al. [38], where their GO nanocarrier did not indicate any cytotoxic activity against the cancer cell lines tested. 

The results have been further tested with t-test statistical analysis for accuracy. No significant difference between all the three tested samples (GAGPAu, PA and GO) was observed in the 3T3 cell lines when compared with the control cells, as revealed by the *p*-value (*p* > 0. 5). However, in the HepG2 cells, the last three doses of PA (6.3, 25 and 100 μg/mL) and the last dose of GAGPAu (100 μg/mL) showed significant toxicity as compared to the control cells. The p-value is *p* < 0.1. 

The high surface area and positively charged surface of the nanocomposites influences the cell penetration either through the low-affinity transmembrane protein pathway or by high-affinity folate receptor. The GO uptake is mostly via clathrin-mediated pathway endocytosis [39]. The results of the cytotoxicity assay have confirmed the nanocomposite has good potential anticancer component in theranostics. 

### 2.8. Magnetic Resonance Imaging Studies

**Figure 7:**
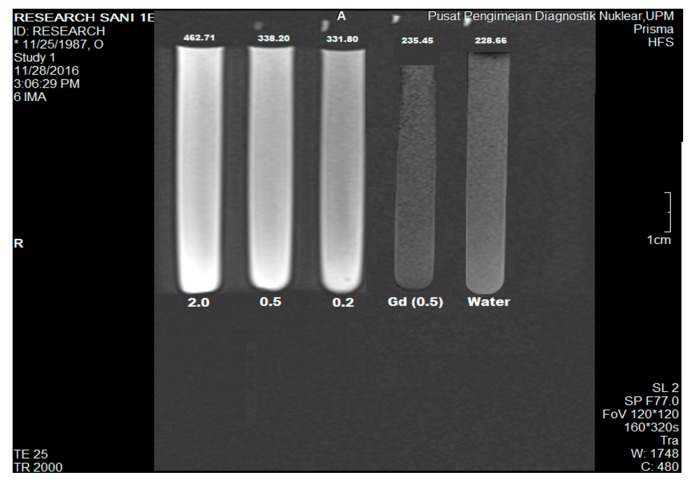
T1−weighted image of protocatechuic acid loaded on GO/Gd nanocarrier after surface coating with gold nanoparticles (GAGPAu) acquired using Prisma 3−Tesla MRI at 2.0, 0.5 and 0.2 *w*/*v* concentrations of Gd^3+^, 0.5 (Gd *w*/*v*) and water reference.

The second theranostic component of the nanocomposite is the diagnostic modality. Magnetic resonance imaging was employed for this purpose. The GOTS was used in the test, which was prepared into various Gd^3+^ concentrations; 2.0, 0.5 and 0.2 *w*/*v*. In addition, two references were used Gd(NO_3_)_3_ (0.5 *w*/*v*) and water. Figure 7 depicts the T1−weighted image of the aqueous GAGPAu nanocomposite distributed in tubes and the references. The steady rise in brightness of the tubes can be visibly seen, which indicates signal enhancement. This is further confirmed by measuring the intensity of each of the tubes, which expectedly shows the values increasing correspondingly with the Gd^3+^ concentrations, 2.0 (452.71), 0.5 (338.20) and 0.2 (331.80) *w*/*v*, Gd 0.5 *w*/*v* (235.45) and water (228.66). The outcome suggests that the developed nanocomposite has higher contrast properties than the conventional Gd-based contrasts. Gd and AuNPs have individually been used for the enhancement of MRI and CT contrasts, respectively [40,41,42,43,44,45,46,47,48,49,50]. Nevertheless, the agents have been combined for bimodal contrast enhancement of MRI/CT [44,51,52,53] as well for the sole purpose of MRI signal improvement using different nanocarriers [7,35]. The MRI signals are believed to be improved through improved interaction between ${\mathrm{Gd}}^{3+}$ ions and GO nanosheets. Moreover, the process is believed to be assisted by the ultrasmall AuNPs at the surface of the GO, which increase the surface area of the nanocomposite, thereby improving water molecular movements within the GO structure [52]. This in turn affects the longitudinal relaxation time (T1 signal) by shortening and reducing the relaxivity, which results in the increase in signal intensity. In addition, the GOTS has an advantage of low toxicity. 

## 3. Materials and Methods

### 3.1. Materials

Graphite flakes (100 mesh size), protocatechuic acid (PA) (98%—170.12 g/mol), phosphate-buffered saline (PBS) potassium permanganate (KMnO_4_) (99%), sulphuric acid (H_2_SO_4_) (98%), hydrogen peroxide (H_2_O_2_) (35%) and ortho-phosphoric acid (H_3_PO_4_) (85%) were purchased from Sigma-Aldrich (St. Louis, MO, USA). Hydrochloric acid (HCl) (37%) and diethyl ether (85%) were supplied by Friedemann Schmidt (Parkwood, WA, USA). Ethyl alcohol (99.7%) was obtained from Hayman. Tetrachloroauric (III) acid trihydrate (49% Au—393.83 g/mol) and gadolinium (III) nitrate hexahydrate (99.9%) were supplied by Acros Organics (Morris Plains, NJ, USA), while human liver hepatocellular carcinoma (HepG2) and normal fibroblast (3T3) cell lines were supplied from the American Tissue Culture Collection (ATCC) (Manassas, VA, USA). Deionized water (DI) was used throughout the experiment.

### 3.2. Characterization

The developed nanocomposites were characterized via different techniques. Powder X-ray diffraction (XRD-6000 diffractometer, Shimadzu, Tokyo, Japan), with CuKα radiation (λ = 1.5406 Å, 40 kV and 30 mA) and scan rate of 0.5° θ/min. Fourier transformed infrared spectroscopy (FTIR) (Thermo Nicolet model Nicolet 6700) was done with a KBr disc. Raman spectroscopy study was done on a UHTS 300 Raman spectrometer (WITec GmbH, Ulm, Germany) at laser excitation wavelength of 532 nm. High resolution transmission electron microscope (HRTEM), Tecnai TF20 X–Twin (FEI, Hillsboro, OR, USA) was used in studying the GO and the nanocomposites structures as well as the drug loading. UV–visible spectroscopy was used for drug release studies and was conducted on a Lambda 35 ltraviolet–visible spectrophotometer (PerkinElmer, Boston, MA, USA). Thermogravimetric analysis (TGA)/differential thermogravimetric (DTG) was done on a TGA/DSC 1HT model (Mettler Toledo, Shah Alam, Selangor, Malaysia) at heating rate of 10 °C/min and nitrogen flow rate of 50 mL/min. 

### 3.3. Graphene Oxide (GO) Synthesis

The improved Hummer’s method was adopted in the preparation of the GO nanocarrier. Briefly, concentrated H_2_SO_4_ (360 mL) and H_3_PO_4_ (40 mL) were mixed and then added to graphite powder (3 g) in a 500 mL beaker. The mixture was stirred for about 5 min for homogeneity. Under stirring at room temperature, the slow addition of 18 g KMnO_4_ was immediately followed. By the end of the KMnO_4_ addition, the temperature of the mixture rose to about 40 °C. The mixture was further stirred for 12 h at 50 °C under dark conditions, then poured into a 400 mL iced DI. Prior to that, the mixture was allowed to cool down to room temperature. Hydrogen peroxide, 3 mL was added to the mixture, which melted the ice. The resultant suspension was filtered and washed via a centrifuge with DI (200 mL) first and subsequently with HCl and ethanol (200 mL each). Finally, the sample was washed with diethyl ether (200 mL), followed by filtering and drying at 40 °C in a vacuum oven [18].

### 3.4. Synthesis of Graphene Oxide—Gadolinium and Protocatechuic Acid Nanocomposite

Synthesis of graphene oxide-gadolinium and protocatechuic acid nanocomposite (GAGPA) was prepared by first dissolving the protocatechuic acid (0.6 g) in aqueous medium (50 mL) by heating/stirring at 40 °C. Then 0.0008 M gadolinium nitrate was added and stirred for 20 min, after which a clear solution was obtained. GO, 0.2 g was then added to the solution. The pH of the mixture was adjusted and maintained at 5.5 using 0.5 M sodium hydroxide solution. The dispersion was allowed to stir for 24 h at room temperature in a dark condition. The slurry obtained was washed 3 times with DI water. Prior to that, the slurry was centrifuged to collect the precipitate. The sample was dried at 40 °C in a vacuum over a period of 48 h.

### 3.5. Synthesis of Gold Nanoparticles on the GAGPA Nanocomposite Surface

The GAGPA nanocomposite (0.15 g) was ultrasonically dispersed in DI (90 mL), HAuCl_4_ (2%, 6 mL) was added to the dispersion under stirring at room temperature. NaOH (0.25 M, 2 mL) was then added to the mixture and was allowed to stir for 24 h under nitrogen atmosphere and heating at 60 °C. The mixture was centrifuged and re-dispersed in DI water (30 mL); NaBH_4_ (1 M, 20 mL) was then introduced and stirred for an hour. The slurry was then centrifuged/washed six times and dried in a vacuum oven at 70 °C for 24 h. The resulting material is gold nanoparticles on the GAGPA nanocomposite surface. 

### 3.6. Drug Loading and Release from GAGPA Nanocomposite

The release pattern and the amount of the anticancer agent loaded into GAGPA nanocomposite was deduced from the data acquired using a Lambda 35 ultraviolet-visible spectrophotometer. The study was done by first dispersing GAGPA (25 mg) in PBS 7.4 and 4.8 (30 mL of each). The tubes were placed in an oil bath shaker, set at 37 °C. The samples were shaken gently; release media (3 mL) was withdrawn and replaced with PBS (3 mL) at predetermined times intervals. The collected media containing the PA that was released from the nanocomposite were analyzed using the UV-Vis spectrophotometer at wavelength maximum (λ*_max_* = 259 nm). 

### 3.7. Cytotoxicity Study

#### 3.7.1. Cell Culture

The purchased standard fibroblast (3T3) and carcinoma (HepG2) cell lines were cultured using RPMI 1640 as culture medium (Invitrogen, New Zealand). 10% fetal bovine serum and 1% antibiotics (penicillin/streptomycin) were used to supplement the culturing medium. The cells were kept and incubated in a humidified chamber at 37 °C and 5% CO_2_ atmosphere. Culture harvest was done via trypsinization. 

#### 3.7.2. Cytotoxicity Evaluation

For the toxicity evaluation, 96-wells were plated with grown cells with 1.0 × 105 density of cells per well, using 100 μL of cell culture medium. A 24 h attachment period was allowed before the addition of the PA, the nanocarrier (GO) and the GAGPAu nanocomposite at varied concentrations. Incubation period followed (72 h). 3-[4,5-Dimethylthiazol-2-yl]-2,5-diphenyltetrazolium bromide (MTT, 5 mg) was dissolved in PBS (2 mL) and distributed into 96-well plates. The cells were incubated at 37 °C, after which formazan product was obtained. DMSO (100 µL) was added to the cells and then shaken. Measurement of the optical densities of the cells at 570 nm was followed. Viabilities of the cells were expressed inform of percentages with the untreated cells as reference. Triplicate experiments and measurements were done all through the experiments for accuracy and mean ± standard deviations were taken. 

### 3.8. Magnetic Resonance Imaging Analysis

Imaging modality test was done on a 3.0 T MRI clinical instrument (3.0 T Siemens Magnetom, Erlangen, Germany). GAGPAu was prepared in three different concentrations (2.0, 0.5 and 0.2), which were based on Gd^3+^ concentration in the samples. Gd(NO_3_)_3_ (0.5 *w*/*v*) and water were used as references. An MRI phantom was used as holder of the samples, which were placed in the magnetic source area. The samples were imaged with the following conditions: TR/TE: (83/9000) 160 × 320 s and field of view (FOV): 120 × 120. Analysis of the T1-weighted images was done with Syngovia MRI software (Syngo MR E11, Siemens, Erlangen, Germany, 2013). 

## 4. Conclusions

In this work, a theranostic nanodelivery system that consists of both therapeutic and contrast agents were simultaneously loaded onto GO nanosheets for imaging and pharmaceutical applications was successfully prepared. The bimodal theranostic nanodelivery system (GAGPAu) was developed from initial synthesis of GO nanocarrier, followed by aqueous doping of Gd^3+^ and protocatechuic acid via hydrogen bonding and π–π interactions (GAGPA). Subsequently, AuNPs were surface coated through electrostatic interactions (GAGPAu). The drug release study showed above 60% protocatechuic acid was released in the acidic PBS medium compared to less than 40% in the alkaline media, suggesting higher drug delivery in the cancer target site. The cytotoxicity studies of the tumor cells further confirmed the drug release study, which showed cancer cell death at 100 μg/mL GAGPAu dose. The GOTS appeared nontoxic to normal cells. The GAGPAu contrast enhancement for imaging modalities was tested with Tesla 3.0 MRI equipment. The T1 weighted image of the GOTS dispersions showed increment in contrast of the nanocomposite to be higher than pure Gd(NO_3_)_3_ and water reference. This preliminary, in vitro result of this research suggests a possible future solution to the highly toxic chemotherapy and diagnosis of cancer diseases. Further studies especially in the in vivo aspect of the GAGPAu are very important for the way forward. 

## Figures and Tables

**Table 1:** Correlation coefficients (R^2^), percentage saturation (%), rate constants (k), and half-life (*t*_1/2_) of protocatechuic acid release at pH 7.4 and 4.8 PBS from GAGPA as derived from the models.

Sample pH	Correlation Coefficients (R^2^)			Percentage Saturation (%)	Rate Constant (k)	*t*_1/2_ (min)
	Pseudo-First Order	Pseudo-Second Order	Parabolic Diffusion			
7.4	0.863	0.992	0.936	50	4.9 × $10^{-3}$	90
4.8	0.563	0.985	0.932	80	2.3 × $10^{-3}$	170

**Table 2:** Decomposition temperature range (T_range_) maximum peak temperature (T_max_) and change in mass (Delta m).

Sample	T_range_ (°C)	T_max_ (°C)	Delta m (%)
GO (A)	71–255	197	57
Protocatechuic acid (B)	122–308	262	88
GAGPA (C)	38–888	200	68
GAGPAu (D)	73–747	537	58
